# Study on the Properties of Ag-Nb_2_AlC Composite as an Electric Contact Material

**DOI:** 10.3390/molecules30040765

**Published:** 2025-02-07

**Authors:** Xiaochen Huang, Hongdi Zhang, Dazhang Wang, Zijue Zhou

**Affiliations:** 1 School of Material and Chemical Engineering, Bengbu University, Bengbu 233030, China; 2 Engineering Technology Research Center of Silicon-Based Materials, Bengbu 233030, China; 3 School of Chemistry and Materials Engineering, Huainan Normal University, Huainan 232001, China

**Keywords:** Ag-MAX, electrical contact material, arc energy, positive streamer

## Abstract

Ag-Nb_2_AlC composite materials with a Nb_2_AlC volume percentage ranging from 10% to 40% were prepared using the spark plasma sintering method. The composite with 10% Nb_2_AlC achieved a high density of 99.2%. The microhardness exhibited a peak value of 84.8 HV at a Nb_2_AlC content of 30%. The conductivity of the composite material decreases linearly with an increase in Nb_2_AlC content, ranging from 0.134 MS·cm^−1^ to 0.086 MS·cm^−1^. A three-dimensional laser scanning microscope was employed to characterize the morphology following arc erosion, and the erosion area was subsequently measured. Results indicated that arc erosion was concentrated on the Ag-20 vol.% Nb_2_AlC composite material, resulting in a smaller circular erosion area. As Nb_2_AlC content increased to 30% and 40%, the arc shifted, leading to an expansion of the erosion area. Notably, the Ag-30 vol.% Nb_2_AlC composite material exhibited the highest arc energy (3.401 kJ). The eroded surface displayed spattered particles and a convex morphology. Additionally, EDS and Raman spectroscopic analyses revealed the formation of Nb_2_O_5_, Ag_2_O, and Al_2_O_3_ on the surface. The arc erosion mechanism was analyzed from an electrical perspective, indicating that the formation of the arc is attributed to the development of a positive streamer and air breakdown within a strongly nonuniform field. Consequently, the surface atoms of Ag-Nb_2_AlC absorb energy, leading to the formation of metal ions that combine with the ionized air to create oxides. This research lays a theoretical foundation for the application of silver-based electrical contact materials.

## 1. Introduction

Electrical contact materials are essential components in a range of electrical and electronic devices, as they significantly influence the performance, reliability, and efficiency of electrical connections [1,2]. A major challenge associated with these materials is arc erosion, which occurs when an electric arc forms between contacts during operation. The process of arc initiation, stable combustion, and arc quenching is a complex physical and chemical process. The interaction time between the electrode and the arc is only a few tens of milliseconds, which is the result of the combined effects of electricity, heat, magnetism, force, and metallurgy. The arc itself is a high-energy, high-temperature plasma, and the elevated temperatures may lead to the melting of the material. Additionally, the forces generated by the arc can cause material spattering, which alters the surface morphology of the material and results in material loss. Furthermore, the composition of the material may change as it absorbs heat, leading to an increase in material resistance. This surface damage can increase resistance and the likelihood of future failures [3,4,5,6]. The arc erosion can lead to the degradation of contact surfaces, ultimately affecting the reliability and longevity of electrical devices [7,8]. The extent of electrical erosion can vary based on material properties and operational conditions, such as current magnitude and the number of switching cycles. Silver-based electrical contact materials are widely employed in various electrical applications, such as circuit breakers and relays due to their excellent electrical conductivity and thermal properties [8,9,10]. However, a notable drawback of metallic silver is its soft texture and low melting point, which make it difficult to withstand high pressure and high temperature. Consequently, incorporating a second phase reinforcing material has emerged as an effective solution [11,12,13].

MAX phases are a class of materials distinguished by their unique combination of metallic and ceramic properties, represented by the general formula M_n+1_AX_n_, where M denotes an early transition metal, A signifies an A-group element (primarily from groups 13 and 14), and X refers to carbon or nitrogen [14,15]. These materials exhibit a range of advantageous properties that make them suitable for various applications, including electrical contacts [16,17]. The exceptional electrical conductivity of MAX phases is crucial for minimizing resistive losses in electrical contacts, which can lead to enhanced performance in electronic devices. This is particularly beneficial in applications where low contact resistance is essential, such as in Ag-based electrical contact materials. Additionally, MAX phases demonstrate remarkable mechanical properties, including high strength and toughness, which contribute to their durability as electrical contacts. Another significant advantage of MAX phases is their resistance to oxidation and corrosion [18]. This characteristic is vital for maintaining the integrity of electrical contacts over time, especially in harsh environments where exposure to moisture and corrosive agents can lead to failure [19]. Ding et al. confirmed that Ag-Ti_3_AlC_2_ exhibits arc erosion resistance comparable to that of traditional electrical contact materials [20]. The stability of MAX phases under such conditions ensures reliable long-term performance, thereby reducing the need for frequent maintenance or the replacement of electrical components. In summary, the integration of MAX phases into electrical contact applications offers several performance advantages, including excellent electrical conductivity, mechanical strength, durability, and resistance to oxidation. These properties render MAX phases a promising choice for enhancing the performance and reliability of electrical contacts in various electronic devices and systems.

The performance advantages of Ag-MAX in electrical contact applications stem from its unique properties that enhance reliability and efficiency in various electronic devices. In the early stage, we systematically studied the arc erosion performance of Ag-Ti_3_SiC_2_, Ag-Ta_2_AlC, and Ag-Ti_3_AlC_2_ composite materials under different conditions and found that the MAX phase has the effect of dispersing the arc [21,22,23]. Here, we use the spark plasma sintering method to prepare Ag-Nb_2_AlC composite materials with a volume fraction of 10–40% to systematically study the effect of Nb_2_AlC content on the arc erosion performance of Ag-Nb_2_AlC composites when the loading voltage is 10 kV. The arc erosion properties of the four-component Ag-Nb_2_AlC composite materials, such as breakdown current, arc energy, arcing time, and erosion area, were characterized and analyzed. Moreover, the surface morphology and composition of the eroded Ag-Nb_2_AlC composite material were analyzed. We illustrated how the arc occurs under the action of a strongly nonuniform field from an electrical engineering point of view, which helps to understand the arc erosion mechanism of Ag-Nb_2_AlC composites and facilitates scholars to better understand the reasons for the generation of the surface products after arc erosion, as well as the role of the oxide generation on the silver-based electrical contact materials. The arc erosion mechanism provided a theoretical basis for the application of Ag-MAX electrical contact materials.

## 2. Results

### 2.1. Physical Properties

**Figure 1:**
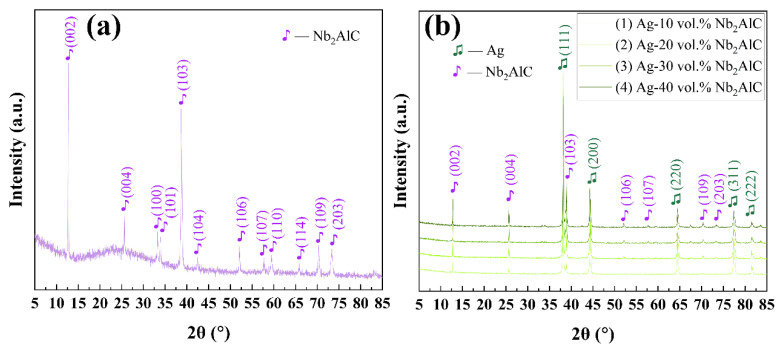
XRD patterns of (**a**) the Nb_2_AlC raw powder and (**b**) Ag-Nb_2_AlC mixtures.

The X-ray diffraction analysis of raw Nb_2_AlC powder (JCPDF: 30-0033) was shown in Figure 1a. In order to facilitate the comparison of the changes in XRD before and after sintering, the mixture of Ag and Nb_2_AlC with different compositions was examined, as shown in Figure 1b. The 2θ values of 12.775, 25.803, 33.368, 33.999, 38.727, 42.614, 52.281, 57.954, 59.530, 65.939, 70.562, and 73.504, which belong to the Nb_2_AlC material, have been detected in Figure 1.

**Figure 2:**
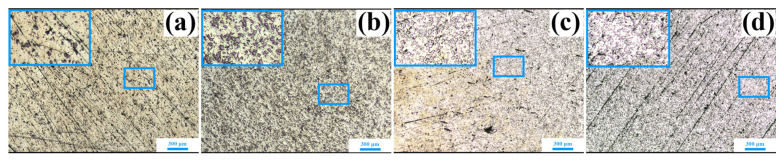
Optical micrographs of the bulk samples of (**a**) Ag-10 vol.% Nb_2_AlC; (**b**) Ag-20 vol.% Nb_2_AlC; (**c**) Ag-30 vol.% Nb_2_AlC; and (**d**) Ag-40 vol.% Nb_2_AlC.

The optical images of the Ag-Nb_2_AlC composites with varying compositions are presented in Figure 2. To more effectively illustrate the uniform distribution of the reinforcement phase on the Ag matrix surface, the local area of each component in Figure 2 has been further enlarged, as shown in the blue boxes. The area within the larger blue rectangle provides an enlarged view of the region within the smaller blue rectangle. From these four enlarged images, it is evident that the gray phase is uniformly distributed throughout the matrix. Notably, Figure 2c, d exhibits some holes, which may have resulted from the grinding and polishing process. This observation suggests that as the Nb_2_AlC volume fraction increases to 30% and 40%, there is a corresponding decrease in performance.

**Figure 3:**
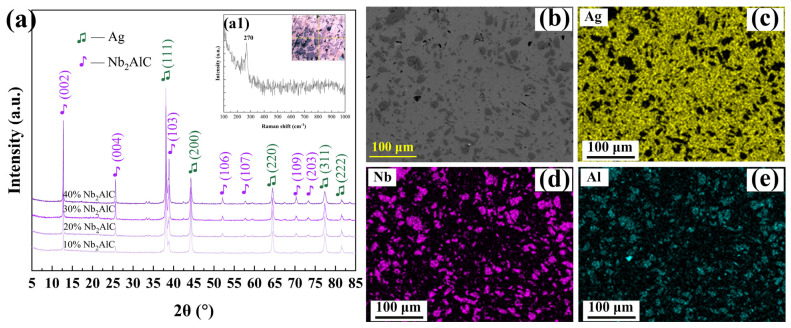
(**a**) XRD pattern of Ag-Nb_2_AlC composite material; (**a1**) Raman spectrum of Ag-Nb_2_AlC; (**b**) SEM image of Ag-20 vol.% Nb_2_AlC; (**c**–**e**) map scanning results of (**b**).

To investigate potential reactions between Ag and Nb_2_AlC following SPS, the composition of the four-component Ag-Nb_2_AlC bulk material was analyzed, as illustrated in Figure 3. As shown in Figure 3a, only two components of the four-component bulk material were detected, which were Ag and Nb_2_AlC. In addition, Raman spectroscopy of the material shown in Figure 3(a1) detected a peak at 270 cm^−1^ for the gray phase illustrated in Figure 2, which corresponds to the Nb_2_AlC phase [24,25]. This observation confirms that no reaction occurred between the two-phase materials during sintering. Additionally, the Ag-20 vol.% Nb_2_AlC composite was subjected to map scanning using a thermal field emission scanning electron microscope (TFE-SEM, ZEISS Gemini 500, Carl Zeiss AG, Oberkochen, Germany), with the results presented in Figure 3b–e. These results further verify that only the two-phase materials, Ag and Nb_2_AlC, are present in the bulk material. The map scanning results indicate that the Nb_2_AlC material is uniformly distributed within the Ag matrix.

**Figure 4:**
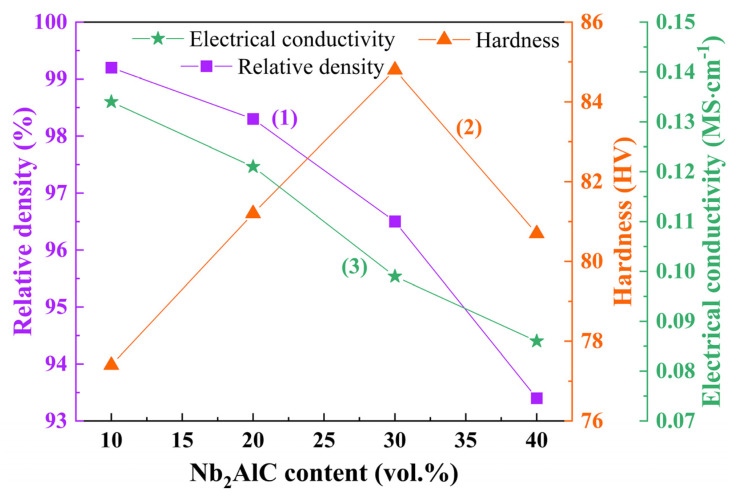
Physical property curves of Ag-Nb_2_AlC composite materials.

The physical properties of the Ag-Nb_2_AlC composite are illustrated in Figure 4. Curve (1) in Figure 4 demonstrates that when the volume percentage of Nb_2_AlC reaches 10%, the density attains a maximum value of 99.2%. However, as the Nb_2_AlC content increases, the density gradually declines, ultimately reaching a minimum of 93.4%. With the increase in enhanced phases, the interface between the enhancement and the matrix increases and more holes are generated, as shown in Figure 2, so the increased pores cause the density of the material to decline. In curve (2) of Figure 4, the microhardness values of Ag-Nb_2_AlC containing 10–40 vol.% Nb_2_AlC are 77.4 HV, 81.2 HV, 84.8 HV, and 80.7 HV, respectively. The microhardness of the Ag-Nb_2_AlC composite material peaks at 30% Nb_2_AlC content, reaching 84.8 HV. This increase in microhardness can be explained by the contrast in hardness between Ag, which is relatively low, and Nb_2_AlC, which is significantly higher; thus, the microhardness exhibits an upward trend as Nb_2_AlC content increases from 10% to 30%. At 40% Nb_2_AlC content, a slight decrease in hardness is observed. This decline can be attributed to the increased interface and porosity associated with the rising proportion of the reinforcing phase Nb_2_AlC, resulting in a reduction in material density and, consequently, microhardness. Furthermore, curve (3) in Figure 4 indicates that the electrical conductivity of Ag-Nb_2_AlC composites decreases from 0.134 MS·cm^−1^ to 0.086 MS·cm^−1^ as the Nb_2_AlC content increases. This reduction is due to the fact that silver possesses higher electrical conductivity compared to Nb_2_AlC; therefore, as the proportion of Nb_2_AlC increases, the overall electrical conductivity of the Ag-Nb_2_AlC composite materials diminishes.

### 2.2. Arc Erosion Properties of Ag-Nb_2_AlC Composites

**Figure 5:**
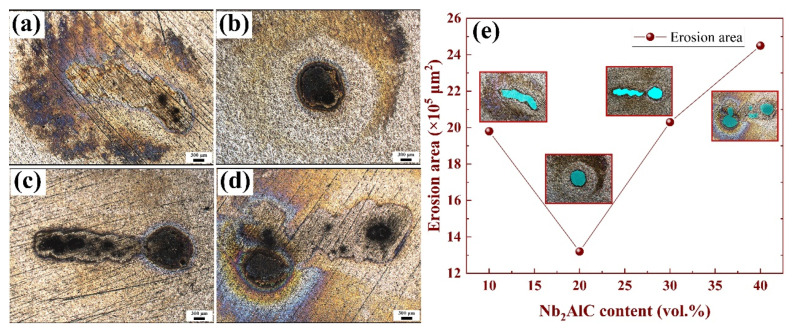
Surface morphology of materials subjected to 10 kV voltage erosion. (**a**) Ag-10 vol.% Nb_2_AlC; (**b**) Ag-20 vol.% Nb_2_AlC; (**c**) Ag-30 vol.% Nb_2_AlC; (**d**) Ag-40 vol.% Nb_2_AlC; and (**e**) the erosion area of Ag-Nb_2_AlC composites.

Under the influence of 10 kV, arc erosion experiments were conducted on Ag-10–40 vol.% Nb_2_AlC composite materials. The eroded surface is depicted in Figure 5, which was captured using a three-dimensional laser scanning microscope. The image reveals that with a Nb_2_AlC content of 10%, the erosion area is relatively shallow, characterized by a black center and colored edges. As the Nb_2_AlC content increases to 20%, the erosion becomes more concentrated and takes on a circular shape. When the Nb_2_AlC content reaches 30% and 40%, similar circular regions are observed in Figure 5c,d, leading us to hypothesize that the arcs in Figure 5c,d originate from these circular areas before gradually migrating to other locations. This process occurs rapidly, completing within 40 ms. Unfortunately, there is currently no technology available to capture this phenomenon. To accurately assess the size of the eroded area, measurements were taken using the software associated with the three-dimensional scanning laser microscope. The test areas are illustrated in the four images of Figure 5e, marked in blue. As shown in Figure 5e, the erosion area is maximized at a Nb_2_AlC content of 40%, while it is minimized at a content of 20%.

**Figure 6:**
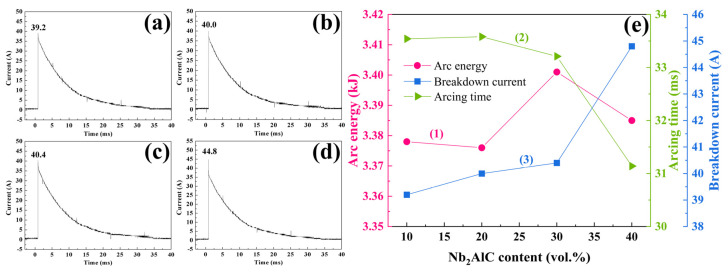
Current–time curves of (**a**) Ag-10 vol. % Nb_2_AlC; (**b**) Ag-20 vol. % Nb_2_AlC; (**c**) Ag-30 vol.% Nb_2_AlC; and (**d**) Ag-40 vol.% Nb_2_AlC materials. (**e**) Graphs illustrating the relationship between arc energy, arcing duration, and breakdown current of Ag–Nb_2_AlC composites.

The current–time curves of Ag-Nb_2_AlC composites are presented in Figure 6a–d. From these figures, it is evident that the current reaches its peak value instantaneously at the moment of arc discharge, followed by a brief period during which the current gradually declines to zero. In curve (3) of Figure 6e, it is observed that the breakdown current increases progressively with higher Nb_2_AlC content, reaching a maximum value of 44.8 A. Additionally, curve (2) of Figure 6e indicates that the arcing time of Ag-Nb_2_AlC composites ranges from 31.14 to 33.54 ms, which decreases gradually as the Nb_2_AlC content increases. This duration is shorter than that of the Ag-Ta_2_AlC composite with the same composition [22]. The arc energy for Ag-Nb_2_AlC composites with four different compositions was calculated using Equation (1) [26], and the results are depicted in curve (1) of Figure 6e.

(1) $$E=\int_{0}^{t_{a}}Uidt$$

In this context, E represents the arc energy in joules (J), *U* denotes the loading voltage in kilovolts (kV), *i* signifies the breakdown current in amperes (A), and *t_a_* indicates the arcing time. As illustrated in Equation (1), when the loading voltage is held constant, the arc energy is closely linked to both the arcing time and the breakdown current. The calculated results for arc energy are depicted in curve (1) of Figure 6e. Notably, the arc energy reaches a maximum value of 3.401 kJ when the Nb_2_AlC content is at 30%. Overall, the arc energy of Ag-Nb_2_AlC composites across four different compositions ranges from 3.378 kJ to 3.401 kJ.

**Figure 7:**
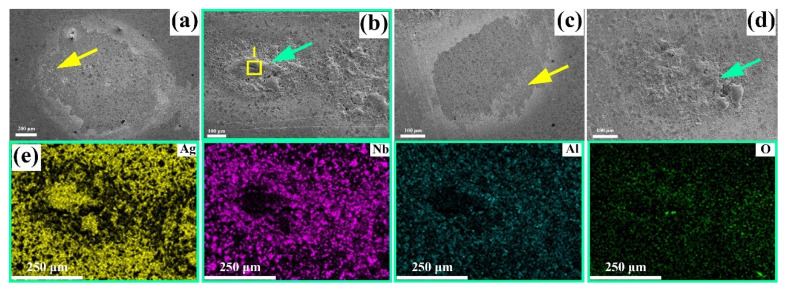
(**a**,**b**) Eroded surface of Ag-30 vol.% Nb_2_AlC composite; (**c**,**d**) eroded surface of Ag-40 vol.% Nb_2_AlC composite; (**e**) map scanning spectrum of (**b**).

Based on the high arc energy of the Ag-30 vol.% Nb_2_AlC and Ag-40 vol.% Nb_2_AlC composites, the morphology of the material after erosion was analyzed through micro-morphological examination, as illustrated in Figure 7a–d. The arrows in Figure 7a,c indicate the morphology of the spattered particles produced during erosion, while the arrows in Figure 7b,d denote the morphology associated with melting and solidification following erosion. The topography in Figure 7b was analyzed using map scanning, which detected the presence of Ag, Nb, Al, and O elements, with their distribution depicted in Figure 7e. It is evident that the protruded morphology in Figure 7b primarily consists of the Ag element. This is attributed to the relatively low melting point of Ag, which is only 961.8 °C, making it susceptible to forming a molten region during high energy and high heat of arc erosion. Subsequently, as the arc extinguishes, the molten Ag cools and solidifies, resulting in the morphology observed in Figure 7b.

**Figure 8:**
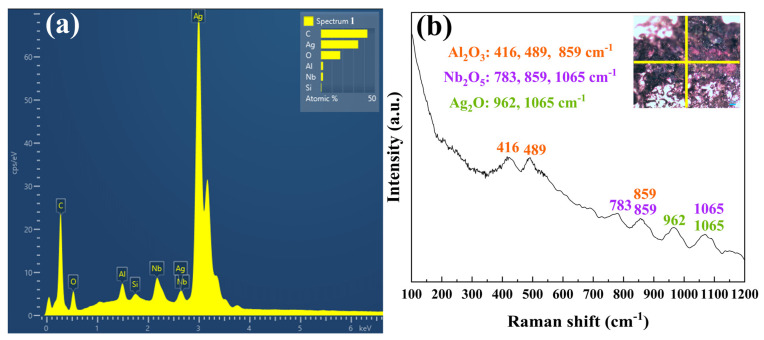
(**a**) Point scanning result of rectangle 1 in Figure 7b; (**b**) Raman spectroscopic result of eroded surface of Ag-Nb_2_AlC composite.

Point-scanning analysis was conducted on the position of rectangle 1 in Figure 7b, with the results presented in Figure 8a. The figure indicates that the concentration of Ag is elevated at this position, along with the detection of trace amounts of Al, Si, Nb, and O. The presence of Si may be attributed to the polishing process, while the detection of O suggests that oxides may have formed during the arc discharge process. To further investigate this hypothesis, Raman spectrum analysis was performed on the eroded surface. The laser used for the Raman spectrum was directed at the intersection of the yellow line in Figure 8b. The results of the Raman spectrum are depicted in Figure 8b, revealing peaks corresponding to the compound Al_2_O_3_ (R040096, R060020, X050045). Additionally, peaks associated with Nb_2_O_5_ and Ag_2_O were also identified [27,28,29,30], with a shift at 859 cm^−1^ corresponding to both Al_2_O_3_ and Nb_2_O_5_ and a shift at 1065 cm^−1^ associated with both Ag_2_O and Nb_2_O_5_. Overall, the Raman spectrometer confirmed that the eroded Ag-Nb_2_AlC surface did produce oxides of Al, Ag, and Nb, which aligns with the point scanning results shown in Figure 8a.

## 3. Discussion

**Figure 9:**
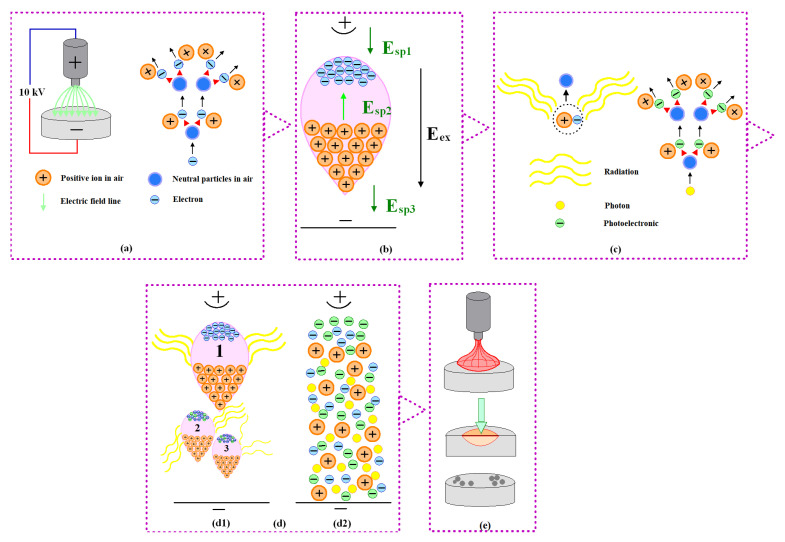
Schematic diagram of (**a**) ionization by collision; (**b**) electron avalanche; (**c**) photoionization; (**d1**) secondary electron avalanche; (**d2**) breakdown of gas; (**e**) arc erosion.

The arc erosion experiment is conducted in air. While air is generally considered an insulator, it can conduct electricity under certain external conditions, such as radiation and voltage. In this experiment, the cathode is made of Ag-Nb_2_AlC composite material and is shaped like a disk, while the anode is a tungsten rod with a pointed tip. When a voltage of 10 kV is applied between the two electrodes, a strongly nonuniform field is generated. A small number of charged particles originally presented in the air were accelerated by the electric field. Ionization energy can be defined as the energy required for a gaseous atom in the basal state, which can lose an electron and become a gaseous cation by overcoming the attraction of the nuclear charge to the electron. Electrons could move toward the anode, colliding frequently with gas particles. If the gas particles received more energy than the ionization energy during collision, a large number of electrons and positive ions would be produced. The newly created electrons moving toward the anode could similarly collide with the gas particles; thus, electrons and positive ions are created again. The number of charged particles increases sharply, ultimately resulting in the formation of a primary electron avalanche, as illustrated in Figure 9a,b.

Under the influence of an extremely uneven electric field, denoted as E_ex_, electrons and positive ions migrate toward the cathode and anode, respectively, resulting in the generation of electric fields E_sp1_ and E_sp3_. The migration rate of the electrons was faster than that of the positive ions; consequently, the electrons accumulated at the head of the primary electron avalanche, while the positive ions remained in their original positions at the time of generation. This disparity leads to the formation of an internal electric field, denoted as E_sp2_, which is oriented opposite to E_ex_, as illustrated in Figure 9b. Within the primary electron avalanche, E_sp2_ promotes a strong combination of electrons and positive ions. The molecules or atoms return to their base state, during which many photoelectrons are released. Each photon possesses a certain amount of energy, which is responsible for generating electrons, as illustrated in Figure 9c. Under the influence of the electric field, the photoelectrons continue to collide with particles, producing additional positive ions and electrons. This results in a secondary electronic avalanche, where the electrons from this secondary collapse enter the region of the primary electronic avalanche. The process of secondary electronic collapse, along with the continuous merging of the primary electronic collapse channel, is referred to as positive streamer [31], as depicted in Figure 9d. This process highlights the cascading effect of photoionization, where the initial release of electrons can lead to a significant increase in the number of charged particles through continued collisions and interactions. The electrons at the head of the secondary electron avalanche entered the space charge region at the head of the primary electron avalanche, where most of the electrons formed negative ions. A significant quantity of particles carrying both positive and negative charges can create a state of matter known as plasma. This specific phenomenon, referred to as a positive streamer, plays a crucial role in various physical processes. The details of this occurrence can be observed in Figure 9d, which illustrates the formation and characteristics of the positive streamer in a visual format [32]. The transformation from an avalanche to a streamer and the space charge field must increase to a level [33].

After applying a voltage of 10 kV to the W anode and Ag-Nb_2_AlC cathode, as the two electrodes slowly approach, the space charge field gradually increased as the distance decreased. As a result, at a certain distance between the electrodes, a positive streamer was generated. Subsequently, the positive streamer toward the cathode rapidly developed due to the continuous participation of the secondary electron avalanche, as shown in Figure 9(d1). Following this, the positive streamer directed toward the cathode quickly intensified, a process that was significantly aided by the ongoing involvement of the secondary electron avalanche, as illustrated in Figure 9(d2). This enhancement in the conductivity within the gap separating the two electrodes played a critical role in facilitating the breakdown of the gas present in that space. Additionally, the analysis of the current versus time curve revealed a distinct current spike, characterized as breakdown current, which is prominently displayed in Figure 6. This spike serves as a clear indicator of the dynamic processes occurring during the breakdown phase. The gas breakdown was accompanied by considerable noise and very strong light. This discharge was potentially self-sustaining once the positive streamer formed under the action of a strongly nonuniform field [34]. Nitrogen and oxygen demonstrated ionization energies of 15.6 eV and 12.5 eV, respectively [35], and because the gas between the two electrodes became conductive, the energy of the collision exceeded the ionization energy of either oxygen or nitrogen.

The work function of Nb_2_AlC was calculated using first principles. Spin-polarized first-principles calculations were conducted employing the Perdew–Burke–Ernzerhof (PBE) exchange–correlation functional, as implemented in the Vienna Ab Initio Simulation Package (VASP). The work function of Nb_2_AlC was 6.5697 eV. This value represents only half the ionization energy of oxygen or nitrogen. Consequently, Ag-Nb_2_AlC can be decomposed into the corresponding metal ions when subjected to a loading voltage of 10 kV. Notably, the work function of Nb_2_AlC is lower than that of SnO_2_ and Ti_3_SiC_2_ [36,37], with the work function of Ti_3_SiC_2_ measured at 5.07 eV. A lower work function indicates that the material is more easily ionized and capable of generating arcs. Previous studies have demonstrated that the breakdown current of the Ag-30 vol.% Ti_3_SiC_2_ composite material was 51.1 A [21], exceeding the breakdown current of the Ag-Nb_2_AlC composite material, which consists of four components.

The charged particles present in the electron avalanche, positive streamer, and metallic vapor disappeared. Charged particles, mainly electrons in the electron avalanche, flowed into the anode due to the force of the electric field and neutralized the charge. The charged particles diffused from an area of higher concentration to an area of lower concentration. Moreover, the positive and negative charges met (leading to the occurrence of charge transfer) and combined to form neutral particles, known as recombination. During the recombination process, the energy absorbed during the original ionization of the charged particle (ionization energy or work function) will be normally released as a photon, where the greater the relative velocities of the charged particles involved in the recombination, the smaller the probability of recombination. The speed of electrons was significantly greater than that of ions, resulting in a lower probability of electron recombing with a positive ion compared with the recombination of a negative and positive ion. When the arc erosion experiment was performed under atmosphere, the oxygen ions combined with the ionized Ag, Ta, and Al ions between the two electrodes after gas discharge was generated, forming the corresponding oxides, namely Ag_2_O, Nb_2_O_5_, and Al_2_O_3_, as shown in Figure 8. Some studies have confirmed that the addition of Al_2_O_3_ can enhance the hardness and wear resistance of electrical contact samples [38]. The addition of metal oxides to the electrical contact material can effectively improve the wettability between the base material and the oxide, reducing droplet splashing and mass loss [39,40,41,42,43,44,45]. Additionally, the literature indicates that the Al_2_O_3_ generated on the surface absorbs a significant amount of heat during vaporization, which shortens the arc life and effectively enhances the reliability of Ag-Ti_2_AlN electric contact materials [46].

During arc combustion, the production and disappearance of charged particles reached a dynamic equilibrium. If the rate of production of charged particles was less than the rate of disappearance of the charged particles, the current would gradually decrease to zero, as shown in the arcing time of the current–time curve in Figure 6, indicating that the arc was extinguished. Finally, under the combined action of electrostatic force, plasma force, and other forces [47,48], the cathode surface formed a bulge and spattered particles, as shown in Figure 5, Figure 7 and Figure 9e. Similar phenomena have been described in detail in previous studies [49]. When the arc was extinguished, the material cooled and solidified, and these sputtering particles remained on the surface of the cathode, forming morphological features as shown in Figure 5a,b, Figure 7e and Figure 9e.

## 4. Materials and Methods

### 4.1. Sample Preparation

Ag-Nb_2_AlC composite materials were prepared using silver powder and Nb_2_AlC powder. The silver powder was purchased from Sinopharm Chemical Reagent Co., Ltd. (Shanghai, China). Nb_2_AlC powder comes from Laizhou Kai Kai Ceramic Materials Company Ltd. (Laizhou, China). Before sintering, X-ray diffraction analysis (XRD, SmartLabSE, Tokyo, Japan) was performed on Nb_2_AlC powder and Ag-Nb_2_AlC mixed powder. After two hours of mechanical mixing of Ag powder and Nb_2_AlC powder, Ag-Nb_2_AlC composite materials were prepared using spark plasma sintering (SPS) at a pressure of 30 MPa (SPS-3T-3-MIN, Shanghai Chenhua Science Technology Corp., Ltd., Shanghai, China). The heating rate during the spark plasma sintering process was set to 100 °C/min, with a hold time of half an hour at 700 °C. The samples were then allowed to cool to room temperature within the furnace before being removed for grinding and polishing. The sintered composition was then analyzed using XRD and Raman spectroscopy, and the elements were analyzed by a thermal field emission scanning electron microscope (TFE-SEM, ZEISS Gemini 500, Carl Zeiss AG, Oberkochen, Germany) equipped with energy-dispersive X-ray spectroscopy (EDS, Oxford Aztec UltimMax100, Oxford Instruments, London, UK).

The densities of Ag-Nb_2_AlC were determined using the Archimedes’ method. The distribution of enhanced phases within the samples was observed with an optical microscope (LIOO M110T, Beijing Jinghao Yongcheng Trading Co., Beijing, China). Additionally, the microhardness of materials with varying compositions under 100 g for 30 s was assessed using a microhardness tester (YZHV-IZP, Shanghai Aolong Xingdi Testing Instrument Co., Ltd., Shanghai, China). The electrical conductivity was measured employing the four-point probe method (ST2258C, Suzhou Lattice Electronics Co., Ltd., Suzhou, China).

### 4.2. Arc Erosion Test

A self-made arc erosion simulation device was employed to conduct arc erosion experiments on Ag-Nb_2_AlC composite materials with four different compositions. The anode consists of a W rod with a tip, while the cathode is made of Ag-Nb_2_AlC composite material. The specific details of the device are described in ref. [22]. After setting the loading voltage to 10 kV, the cathode and anode were allowed to move slowly relative to each other, gradually approaching one another. At a specific moment, an arc was generated, resulting in erosion of the Ag-Nb_2_AlC composite surface. Subsequently, the arc erosion performance of the eroded surface was characterized, including an analysis of the surface composition and morphology post-erosion. The characterization of arc erosion performance encompassed measurements of breakdown current, arc energy, arcing time, breakdown strength, and the erosion area. Component analysis was conducted using a thermal field emission scanning electron microscope (TFE-SEM, ZEISS Gemini 500, Carl Zeiss AG, Oberkochen, Germany) equipped with energy-dispersive X-ray spectroscopy (EDS, Oxford Aztec UltimMax100, Oxford Instruments, London, England) and a Raman spectrometer (Lab RAM-HR, HORIBA, Kyoto, Japan). The surface morphology was obtained by integrating scanning electron microscopy images and three-dimensional laser scanning morphology (3D LSCM, Keyence VK-X250, Keyence Corporation, Osaka, Japan). The software equipped with the 3D LSCM, Multifile Analyzer 1.0, was used to measure the arc erosion area.

## 5. Conclusions

The Ag-10–40 vol.% Nb_2_AlC composite material was prepared using the spark plasma sintering method, and its physical properties were subsequently measured. The disk-shaped Ag-Nb_2_AlC composite serves as the cathode, while a tungsten (W) rod with a pointed tip is utilized as the anode. A self-made arc erosion simulation device applies a voltage of 10 kV across the two electrodes, creating a strongly nonuniform field that facilitates air breakdown and the formation of positive streamers. This process induces arc erosion on the surface of the Ag-Nb_2_AlC composite, resulting in erosion traces of various shapes, which are characterized to assess the arc erosion performance.

(1) As the content of Nb_2_AlC increases, both the density and electrical conductivity of the Ag-Nb_2_AlC composite material exhibit a decreasing trend. Specifically, the density declines from 99.2% to 93.4%, while the electrical conductivity decreases from 0.134 MS·cm^−1^ to 0.086 MS·cm^−1^. Additionally, the hardness of the material initially increases and then decreases with an increase in the reinforcement phase. The highest recorded hardness of the Ag-30 vol.% Nb_2_AlC composite material is 84.8 HV.

(2) The Ag-20 vol.% Nb_2_AlC composite material exhibited the smallest erosion area, characterized by circular arc erosion traces. Meanwhile, the erosion areas of the Ag-30 vol.% Nb_2_AlC and Ag-40 vol.% Nb_2_AlC composites increased, resulting in irregularly shaped erosion traces.

(3) The arcing time gradually decreases as the Nb_2_AlC content increases, while the breakdown current exhibits an opposing trend. Upon calculation, it is determined that the arc energy of the Ag-30 vol.% Nb_2_AlC composite material is the highest, measuring 3.401 kJ.

(4) The metal on the surface of the Ag-Nb_2_AlC composite material becomes ionized due to the influence of a strongly nonuniform field, subsequently participating in the arc combustion process. The metal ions react with the ionized oxygen atoms to form Nb_2_O_5_, Ag_2_O, and Al_2_O_3_, after which the arc extinguishes.

## Data Availability

Data are contained within the article.
